# Design and manufacture of a soft robot with dual-interaction in virtual reality

**DOI:** 10.1016/j.heliyon.2023.e19997

**Published:** 2023-09-09

**Authors:** Majid Eslami, Mostafa Pirmoradian, Ali Mokhtarian, Shaghayegh Baghaei

**Affiliations:** Department of Mechanical Engineering, Khomeinishahr Branch, Islamic Azad University, Khomeinishahr, Isfahan, Iran

**Keywords:** Virtual reality, Soft robot, Arduino, Unity, Control panel, Bending measurement sensor

## Abstract

This paper examines the design and fabrication of a soft robot that can connect to a virtual reality environment. This study's primary objective is to utilize these technologies concurrently and demonstrate their applicability in various applications, particularly rehabilitation. Therefore, the process of designing and modeling the soft robot is carried out, and an applied model is created using a 3D printer and silicon material, which is then installed on gloves. Using Unity software, a virtual reality environment is created in which programs, commands, and Arduino processors control the movements of the soft robot, allowing the user to move and pick up an object in a real environment while wearing gloves, and to adjust the amount of pressure and angle of its motion based on the size of each virtual object. During the system evaluation phase, a delay in the performance and reaction time of the soft robot installed on the gloves is observed. This delay is reduced by modifying the programming structure, resulting in optimal system functionality. This capability is used to create proper mobility conditions and rehabilitation for the majority of patients with wrist injuries resulting from strokes and accidents, and it may be effective in accelerating patients' recoveries.

## 1 Introduction

Today, robots are found in different applications. There is a growing need for high-speed, robotic assembly of small parts [[1], [2], [3], [4], [5], [6], [7], [8]]. Robots and virtual reality (VR) are currently of paramount relevance to the advancement of humanity [9,10]. Thanks to robotics and VR, humanity has quickly reached a better future. Owing to its illustrious history, robots have always aided people in overcoming difficult and potentially dangerous obstacles, and despite their unique advances in recent years, VR and this technology continue to improve in all disciplines. VR appeared to be tied to the introduction of microprocessors at the end of the 20th century [11] and was widely used in many fields, including educational environments [[12], [13], [14]]. Horváthová et al. [15] have explored the use of VR in psychiatry and the alleviation of anxiety associated with phobias. VR can also affect other scientific fields. Ryan et al. [16] investigated the influence of virtual technology on walking speed. The diverse applications of VR may be one of the most important aspects of this technology that leads to its widespread use and production in medicine and psychiatry. VR has surpassed other medical disciplines in improving treatment possibilities. Lewis Francesca et al. [17], who introduced knowledge of VR into rehabilitation processes, have acknowledged its effectiveness and utility in patients' therapy. The condition of patients with autism has improved as a result of the research of Bellani et al. [18]. In spite of all robotics-related application research, scientists have designed and controlled soft or flexible robots due to the need for a robot structure adaptable to physical conditions and human-like movement. This technology can be found in the construction of origami, which is the Japanese term for flexible paper-based objects [19]. Soft or neo-flex and modular operators are frequently employed in the creation of robots with an origami structure [20,21].

Soft robots have the same number of degrees of freedom as human organs and can do things that other robots can't. Software and computational analysis can also figure out how each organ moves and how much pressure is put on it [22]. Two categories of robot operators have been identified. Sandwich-structured operators that respond to electricity and include memory alloys, current or liquid operators, such as hydraulic and pneumatic actuators utilizing airflow or oil pressure. Depending on their use, the motion structure of soft robots is classified into three kinds [23]. (1) Robots that can only be opened and closed. (2) Soft robots with the ability to bend in addition to opening and closing. (3) Soft robots capable of rotation and bending in addition to opening and closing [[24], [25], [26]].

Usually, materials with elastic and elastomeric coefficients and matrices are used to manufacture flexible and soft robots [27], which is comparable to the composites' properties. Elastomeric materials typically have high elasticity and are extremely useful in the design of elastic mechanisms; however, their mechanical properties must be considered during the design process [28]. Yap et al. [29] investigated one application of soft robots in the rehabilitation of stroke patients and discovered that installing soft robots on gloves increases patients' mobility. The integration of VR and robotics may be one of the greatest achievements of engineers in their efforts to create new, realizable conditions for addressing new challenges in life and science. The combination of robotics and VR has been the subject of a number of studies at colleges and universities, including the investigation of Rose et al. [30] on the application of both technologies in upper body rehabilitation. They assessed the effect of VR and robots on the improvement of patient care. Boian et al. [31] studied the effects of VR and robotics on the rapid treatment of stroke patients utilizing robotic gloves equipped with sensors. During the recuperation phase, the design by Frisoli et al. [32] of a 5-degree-of-freedom rehabilitation robot integrated with VR also demonstrated significant improvement in the treatment process. Drones and other robotic construction technologies were simulated in a virtual reality setting, thanks to the work of da Silva et al. [33]. After analyzing several methods and scenarios for robotic construction simulation methodology, a construction simulation methodology applied to three architectural aspects was presented. In their experiment, they put a building simulation through its paces and then compare the results to those obtained using more conventional techniques, focusing on reducing both construction time and costs. Kutlu et al. [34] demonstrated the efficacy of combining VR and robotics in constructing an upper body robot for patients with stroke. In these investigations, the interaction between the actual robot and the software-based virtual world is crucial, as the robot is a physical construct and VR is software. Combining these two domains of hardware and software requires an electronic circuit to evaluate and process data and commands. The Arduino board, an open-source processor, has been utilized in the majority of academic research and experimentation. There are studies by Kutlu et al. [34], and Guang et al. [35] that demonstrate the application and evolution of this hardware.

Owing to the significance of using soft robots and their versatility and application in rehabilitation and VR as an advancement in medical informatics, this study is done to utilize robotics and VR in a novel manner. Eventually, using the hybrid method and a sophisticated electronic processor put on the gloves and interacting with the virtual environment, it is possible to generate a new possibility to utilize integrated soft robots that result in more practical rehabilitation applications. Based on the size of each object in the virtual environment, the intelligent control of the robot and artificial intelligence capabilities in Unity software make the user more intuitive in the usage of soft robots and enable observation of varied angles in real settings.

## 2 Methods

**Fig. 1:**
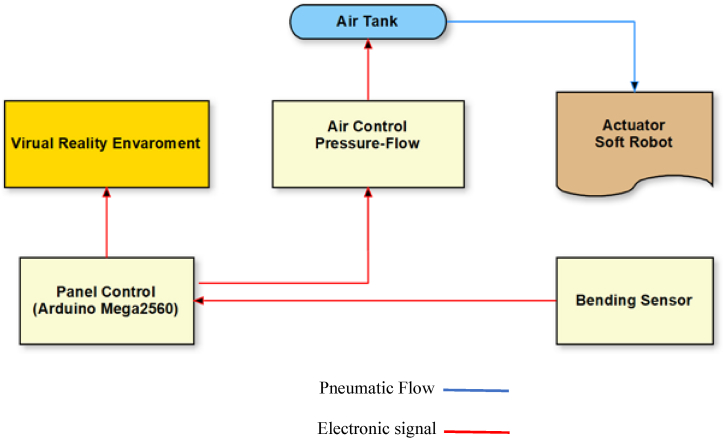
The relationship between soft robot and virtual reality environment.

According to the organization of the research, there are three primary sections. First, the VR environment is designed, then the design and production methods of the soft robot are explained, and lastly, the connection between two sections via a CPU and electronic hardware is described. Important to this research is the evaluation of the soft robot's bending and the transmission of this information to a virtual reality environment. Then, when the size of the object in the virtual reality environment is identified, the soft robot begins to conform to the size of the object. Fig. 1 depicts the relationship between soft robot and virtual reality environment.

### 2.1 Design and simulate virtual reality

In the first section, the virtual reality according to research condition was designed, for which the design pattern of a virtual environment has followed.

• Develop a scenario that is appropriate to the situation
• Designing 3D models in software, depending on the scenario
• Incorporate 3D models into Unity software
• Coding in Unity programming environment
• Extract the executable file according to the type of hardware and software operation environment (Android - Windows)

**Fig. 2:**
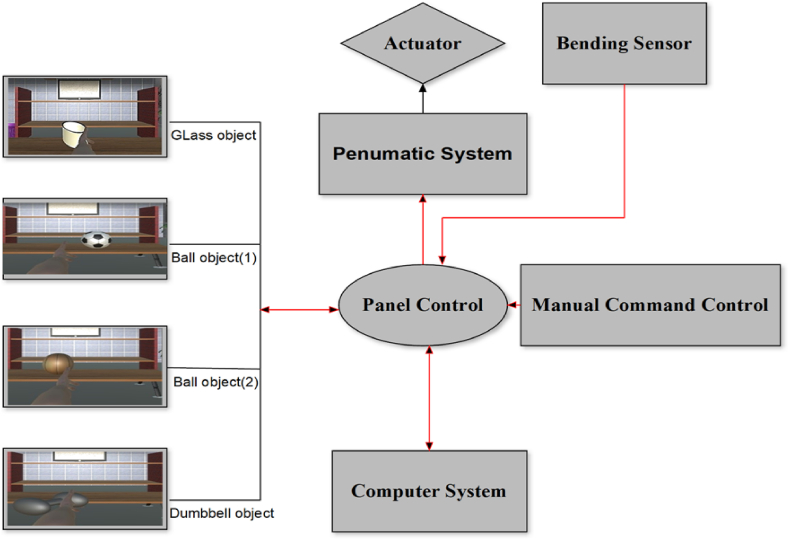
The process of the virtual reality-training phase.

Thus, the virtual reality environment was designed under the Windows operating system and was accessed to the hardware through software coding. A schematic of this environment is shown in Fig. 2.

Fig. 2 depicts four objects, including two distinct balls, one glass, and one dumbbell. The sizes of Ball 1 and Ball 2 are distinct. Thus, we must recognize these sizes and modify the soft robot's bending in the real-world environment based on the size of the object. First created in modeling software, the 3D models were subsequently combined into Game Unity, which is used to build games and VR. In the programming part, tasks such as selecting an item, commanding a serial port, and connecting a processor and a robot were determined by C# and Java definitions. By titling each object, utilizing definitions such as encounters in the Unity engine, and employing artificial intelligence in programming so that each object encounters a virtual hand in the environment, the necessary commands were sent to the soft robot operator via the serial port to activate it, and the bending rate of the soft robot was then modified based on the object name.

### 2.2 Manufacturing a soft robot

**Fig. 3:**
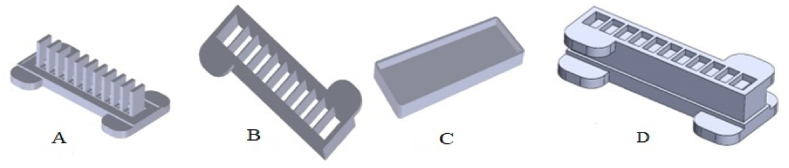
Model and mold of a soft robot used in the research. (A) The first part of the mold (B) The second part of the mold, (C) The underneath part of the mold, (D) The assembled model.

In order to construct a soft robot, a 3D model was first developed and analyzed in CAD software. The schematic of this model is shown in Fig. 3. The required mold for producing the soft robot was made using 3D printing.

**Fig. 4:**
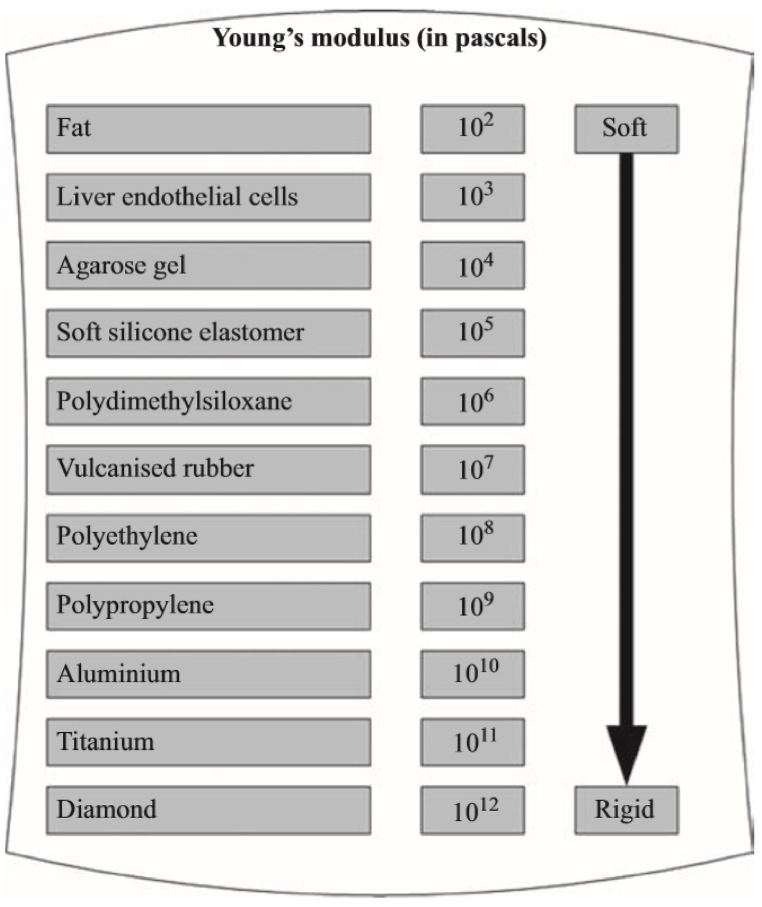
Young's modulus (in Pa) for some engineering and biological materials.

**Fig. 5:**
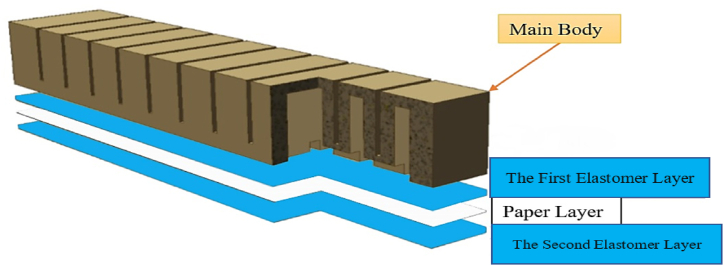
The soft robot's different layers.

After molding using silicon (Elastosil M4601, Young's modulus of MP∼7 MPa, Shore A), like that was used in the study of Mosadegh, Bobak et al. [36] and the hardness of 28 which consists of two sections [37], it was combined with 10: 1 material proportion under laboratory conditions and several models were developed and evaluated (Fig. 4, Fig. 5). For manufacturing the soft robot we must use the soft martial.

**Fig. 6:**
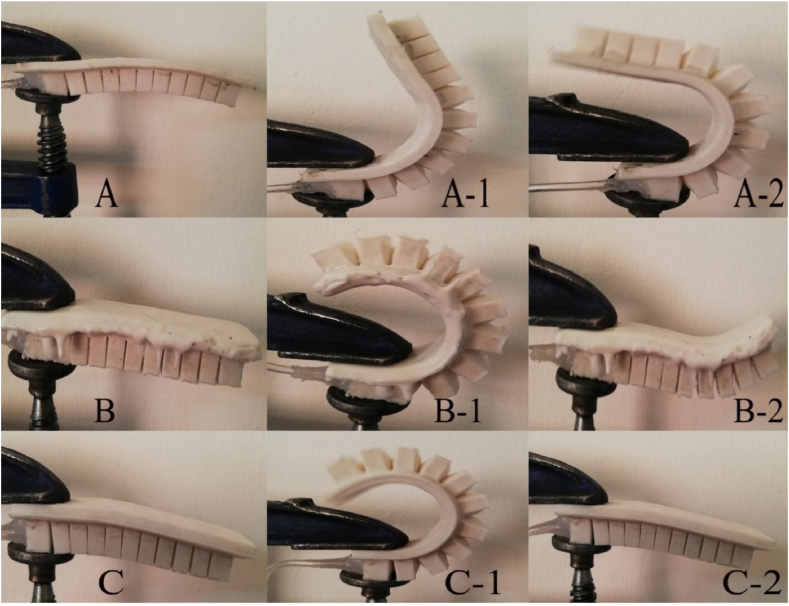
Making multiple models and selecting the best model (C).

Following an evaluation of the surface quality in accordance with the needs of the study being conducted on soft robot gloves, the most suitable model was chosen. (Fig. 6).

**Fig. 7:**
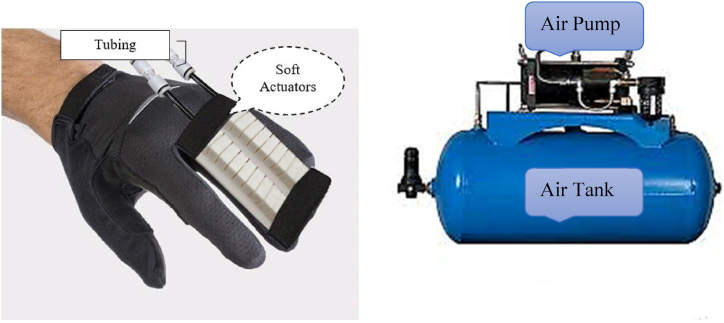
Soft robot installed on gloves (left), and Pneumatic tank (right).

**Fig. 8:**
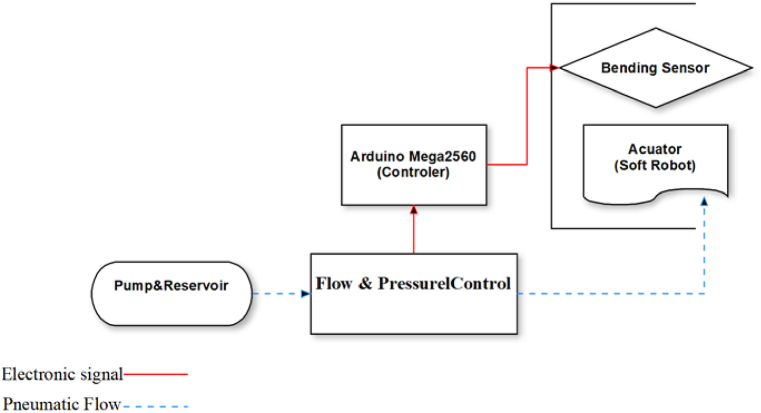
Scheme of the control and command circuit.

Fig. 6 shows that the samples A and B, made with a lower rate of silicon in the air pressure vessel or a lower proportion of silicon in the robot's composition, experienced issues with the robot's bending angle and its ability to return to its initial state. Taking these considerations into account, soft robot prototype C was designed to address the aforementioned issues. After installing the robot on the glove (Fig. 7), the tank and pneumatic structure were constructed according to the robot's generator, and valves and pressure controls were built on the tank due to the pressure effect and controllability of this type of robot via VR. Due to the necessity of safety when using gloves and the variation in the bending angle of the fingers to suit the item, it is conceivable to utilize a bending sensor on the glove to control the angle and flexure of the fingers. Hence, depending on the kind and size of the object in a virtual environment, the user detected differences in the amount of finger bending, and the sensor promptly directed the valve to shut off and the wind direction to reverse when the maximum angle was exceeded. The control circuit is illustrated in Fig. 8.

### 2.3 Design and manufacture of an intelligent control panel

**Fig. 9:**
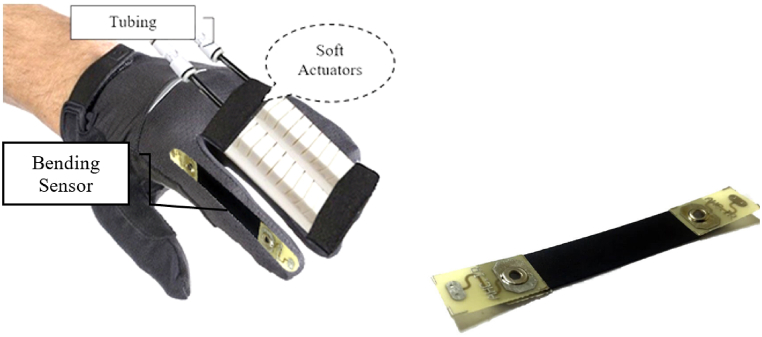
Sensor installed on the soft robot.

After creating the hardware requirements for the project, fabricating and installing the soft robot, the Arduino board MEGA2560 is utilized to process and analyze the project data. Programming in this board's software environment enables the serial protocol connection of VR to the soft robot and solenoid valves. Because to their extensive capabilities, Arduino boards are able to analyze and process many inputs simultaneously. In this study, the angle variations of the robot are measured in relation to the installed bending sensor (which is shown in Fig. 9), and when the robot reaches a certain angular level based on the kind of object, the pressure input tells the robot to cease operation. After attaching this sensor to the micro-ADC port, its resistance is measured, and the robot's bending is analyzed at each instant. As shown in Fig. 9, the installation location of the sensor is under the soft robot and on the fingers so that it measures the degree of bending of the fingers and does not restrict movement.

**Fig. 10:**
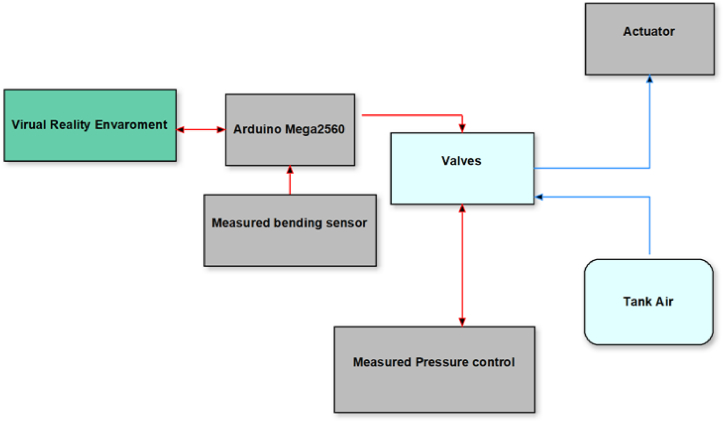
Schematic of the control and command circuit.

We embedded Belding Sensor on glove and Sensor have direct contact to soft robot. The scheduling of setting was that whenever the user wore the gloves, the image of the designed virtual reality environment was displayed on the screen via electronic and pneumatic connections. Afterward, by clicking, the process of moving the body begins sending required instructions to the Arduino board and pneumatic valve and then stimulating the soft robot to provide enough pressure to bend the fingers and as a result, the fingers bend regarding pneumatic pressure. At the same time, being reached to the specific angular level, a resistant sensor that measures the bending angle, disconnects the pneumatic circuit system and returns the robot to its original state. The schematic and structure of the command and control circuit are shown in Fig. 10.

After connecting the virtual environment to the soft robot via a processor and intelligent control panel, under appropriate laboratory conditions, the user observed the amount of bending angle and the force applied to the fingers several times to determine the efficient performance of the robot and VR. Due to the available processor, the system had delays in command, which was resolved by optimizing the code and reducing the delays in programming loops.

## 3 Results

**Fig. 11:**
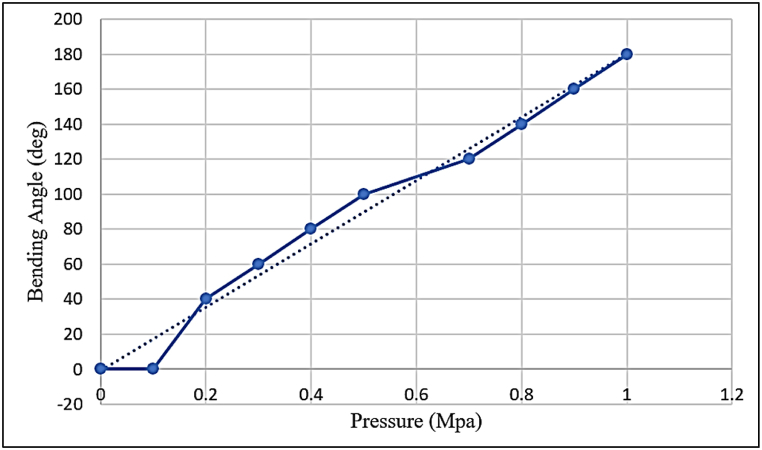
The ratio between inlet pressure and bending angle.

Because of the scope of this research, the findings are analyzed with regard to both the field of VR and that of soft robotics. In the section on soft robots, the ratio of pressure and force that is delivered to the hand as well as the amount that the fingers flex is monitored in order to determine how a user would interact with the conditions that are present in a virtual world. As can be seen in Fig. 11, the bending gauge sensor mounted on the glove measures the bending angle of the soft robot at a variety of different inlet pressures.

**Fig. 12:**
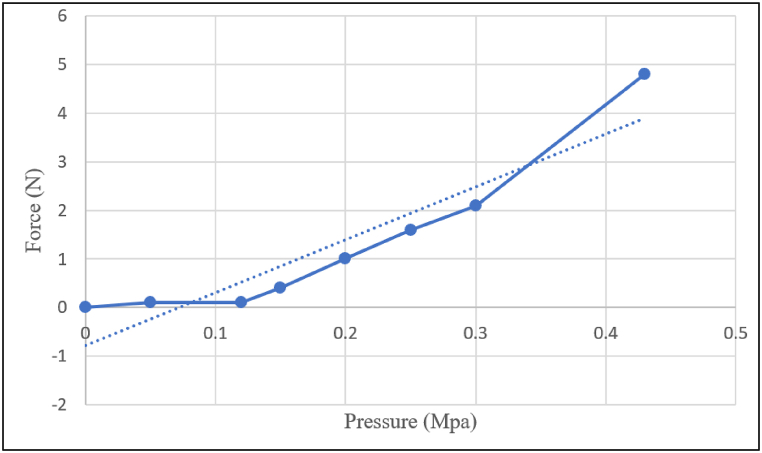
The ratio between input pressure and force.

According to the relationship between force and pressure P=F/A (shown in Fig. 12), the level of bending and force produced by the robot is related to the level of pressure applied to the robot.

**Fig. 13:**
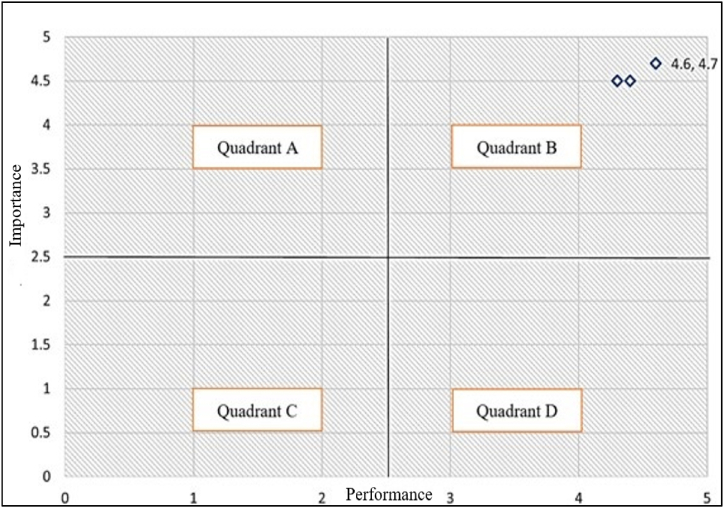
Diagram of user satisfaction assessment.

As indicated previously, delays were eliminated at the beginning of the test condition and after the VR environment instruction was delivered to the system hardware by optimizing code and decreasing loops in Arduino programming, and the system returned to a normal and concurrent state. In addition, measurements of bending sensors are emphasized when programming in the Arduino software environment in order to manage the degree of bending of a soft robot based on the user [38]. Given that the VR assessment criterion is service-dependent, this sort of evaluation has been examined using the Martilla and James criteria [38], and the findings are derived from the software's effectiveness baseline score of five. In the Martilla and James criteria, there are four separate regions; the first one has a high level of satisfaction, while the second one has a low level of satisfaction but a high level of importance. The third area has a low importance rating and a poor satisfaction level, whereas the fourth area is characterized by low importance and high satisfaction. On the basis of this criterion, it is possible to ascertain the effectiveness of this strategy, as well as measure the degree to which customers are pleased with the functional aspects of the product and the services (Fig. 13).

## 4 Conclusion

Design and manufacture of the soft robot and VR environment, as well as their interaction, which is the challenge of these new technologies, were explored in the present study. Modeling a VR world required design and programming using the Unity software package. The soft robot was created using 3D printers and silicon. Using an Arduino MEGA2560 signal processing board, the soft robot was controlled and integrated into a virtual environment. Conditions for testing and evaluation were supplied, and the user evaluated the physical and interaction settings by clicking the mouse in a virtual environment to transmit commands to the pneumatic valve while wearing gloves. The pressure was then applied to move the robot in accordance with the size of the object in the virtual world, and it began to conform to the angles of human fingers.

The glove-mounted sensor displayed the bending angle of the user's fingers in a virtual environment and on the display of the intelligent control panel. The system error based on the synchronization of the virtual environment and the reaction of the soft robot was minimized by programming and reducing the number of code lines, and the robot's response time was ultimately optimized. The findings of the design review based on the Martial and James criteria revealed that users were pleased with the offered equipment, which can be utilized in the medical area to aid in the treatment and recovery of patients with stroke and finger injuries. In addition, the results suggested that the psychological impact of VR and the exceptional capability of flexible and soft robots could aid in the rehabilitation and speedy recovery of patients.

## Author contribution statement

M. Eslami; M. Pirmoradian; A. Mokhtarian; S Baghaie: Conceived and designed the experiments; Performed the experiments; Analyzed and interpreted the data; Contributed reagents, materials, analysis tools or data; Wrote the paper.>

## Data availability statement

No data was used for the research described in the article.

## Declaration of competing interest

The authors declare that they have no known competing financial interests or personal relationships that could have appeared to influence the work reported in this paper.
